# Comparative transcriptome analysis unveiling reactive oxygen species scavenging system of *Sonneratia caseolaris* under salinity stress

**DOI:** 10.3389/fpls.2022.953450

**Published:** 2022-07-25

**Authors:** Yan Zhou, Lizhen Wen, Lixian Liao, Shuangmei Lin, Enting Zheng, Yin Li, Ying Zhang

**Affiliations:** Mangrove Institute, Lingnan Normal University, Zhanjiang, China

**Keywords:** *Sonneratia caseolaris*, reactive oxygen species, antioxidant, transcription factor, salt stress

## Abstract

Many mangrove forests have undergone major changes as a result of human activity and global climate change. *Sonneratia caseolaris* is a common tree located in inner mangroves, and its range extends inland along tidal creeks, as far as the influence of salinity extends. This study investigated the physiological and molecular response mechanisms of *S. caseolaris* by analyzing its antioxidant defense capacity, including its differentially expressed genes (DEGs) under similar salt stress conditions. Salt treatment significantly affected the osmoprotectants and lipid peroxidation in *S. caseolaris* seedlings, which increased proline (Pro) content by 31.01–54.90% during all sample periods and decreased malonaldehyde (MDA) content by 12.81 and 18.17% at 25 and 40 days under 3.0% NaCl treatment. Antioxidant enzyme activities increased significantly following 3.0% NaCl treatment. Transcriptome analysis following *De novo* assembly showed 26,498 matched unigenes. The results showed that 1,263 DEGs responded to transcription factors (TFs) and plant phytohormones and mediated oxidoreductase activity to scavenge reactive oxygen species (ROS) in the control vs. 3.0% NaCl comparison. In addition, the transcription levels of genes associated with auxin and ethylene signal transduction also changed. Under salt stress, ROS scavenging genes (*POD*, *CAT*, and *APX*) and part of AP2, MYB, NAC, C2C2, bHLH, and WRKY TFs were upregulated. This study identified important pathways and candidate genes involved in *S. caseolaris* salinity tolerance and provided suggestions for further research into the mechanisms of salt tolerance in *S. caseolaris*.

## Introduction

Soil salinization has had severe impacts on both agricultural production systems and sensitive ecosystems. More than 6% of the world’s land area, including approximately 20% of all irrigated land, is increasingly being affected by salt accumulation (Munns and Tester, 2008). Salt stress severely damages plant development, resulting in agricultural losses and serious deterioration of plant ecosystems (Ismail and Horie, 2017). High concentrations of sodium in the soil limit water uptake and nutrient absorption (Gong, 2021). Salt stress, caused by this highly saline soil, is one of the most harmful environmental stressors and simultaneously causes ionic toxicity, osmotic stress, and oxidative stress (Tanveer and Yousaf, 2020). These stressors damage cellular membranes, proteins, and nucleic acids, obstruct the photosynthetic system and metabolic function, and inhibit plant growth (Zelm et al., 2020). These studies contribute to the understanding of plant responses to soil salinity stress, which will assist in improving crop stress resistance and yield.

Plants have developed specialized strategies to cope with salt stress, including the accumulation of osmoregulatory substances, such as proline, amino acids, polyamines, quaternary ammonium compounds, and sugars synthesized by certain metabolic pathways to alleviate hyperosmotic stress damage (Zulfiqar et al., 2020). Osmotic stress and subsequent ionic stress are induced by high salinity, resulting in oxidative damage to plant cells due to the excessive accumulation of reactive oxygen species (ROS) (Hasanuzzaman et al., 2021).

Plants are protected from oxidative stress damage by their antioxidant system that detoxifies ROS and maintains the balance of ROS formation under salt stress (Sachdev et al., 2021). In plants, this system includes both enzymatic and non-enzymatic systems. The major antioxidant enzymes are superoxide dismutase (SOD), peroxidase (POD), catalase (CAT), ascorbate peroxidase (APX), and glutathione reductase (GR). The non-enzymatic components include glutathione, ascorbic acid (AsA), tocopherols, thiols, and carotenoids (Sies et al., 1992; El-Shabrawi et al., 2010). Previous studies have reported that there are a separate set of plant hormones and genes involved in ROS scavenging systems that protect plants against salt stress (Hasanuzzaman et al., 2021). By modulating ROS production and metabolism, ethylene functions as a signaling molecule and a key modulator of plant stress responses (Jahan et al., 2021). During abiotic stress adaptation, major ethylene signaling factors such as the gene family AP2/ERF have complex regulatory roles in plants (Tao et al., 2015). Transcription factors, such as AP2/ERF, MYB, WRKY, bZIP, bHLH, C2H2, and NAC, improve salt tolerance by regulating the antioxidant defense mechanism to scavenge ROS (Guo et al., 2014; Xie et al., 2018; Agarwal et al., 2019; Li et al., 2019, 2021a; Zhang et al., 2020a,^2021^, ^2022^; Liu et al., 2021a).

In recent years, the molecular foundation of plant responses to abiotic stress have been extensively studied using high-throughput sequencing technologies, particularly RNA sequencing (RNA-seq) (Li et al., 2021b,c). Recently, RNA-seq has been used to determine dynamic response mechanisms in mangrove plants, such as *Sonneratia alba* (Chen et al., 2011), *Millettia pinnata* (Huang et al., 2012), and *Ceriops tagal* (Xiao et al., 2016) under abiotic stress. A comparative transcriptome study of *S. alba* treated with 0, 250, and 500 mM NaCl has recently been reported (Feng et al., 2020; Wang et al., 2022). *Sonneratia* is a typical mangrove genus with six species and four hybrid taxa that are widely dispersed over the Indo-West Pacific (Yang et al., 2016a; Zhong et al., 2020). *Sonneratia caseolaris* is a medium to large evergreen tree with simple elliptic to narrowly ovate or obovate leaves and a highly specialized root system with multiple root types (Tomlinson, 1994; Zhong et al., 2020). It is common in inner mangroves and extends inland along tidal creeks as far as the influence of salinity extends and occurs naturally in upstream estuary zones in lower saline areas with deep muddy soil, which allow this species to grow in brackish to freshwater environments (Tatongjai et al., 2021). Globally, it has a distribution throughout Southeast Asia, the Malay Archipelago, and the Philippines, China Hainan, Solomon Islands, New Hebrides, and Australia (Tomlinson, 1994). In China, *S. caseolaris* is naturally distributed on Hainan Island and was introduced to northern, colder areas such as Shenzhen and Leizhou in Guangdong Province and Qinzhou and Fangchenggang in Guangxin Province. The species played an important role in mangrove conservation and afforestation in China (Yang et al., 2021). Previous studies on *S. caseolaris* have focused on the biological chemistry (Ebrahim et al., 2012), genetic diversity (Yang et al., 2016a), molecular mechanisms under chilling stress (Yang et al., 2021), photosynthetic characteristics and energetic cost (Li et al., 2016), and comparative anatomy under salt stress (Tatongjai et al., 2021). However, few studies have been reported on the physiology and molecular mechanisms of *S. caseolaris* under abiotic stress.

In this study, we evaluated the soil salt tolerance of *S. caseolaris* and investigated its physiological response to salinity stress. A high-quality *De novo* transcriptome of *S. caseolaris* was used to study gene expression under the same level of salt stress. The results of this study elucidates the mechanisms underlying *S. caseolaris* salt tolerance and provide a valuable genetic resource for further research on mangrove plants.

## Materials and methods

### Plant materials and salt treatments

Seeds of *S. caseolaris* were harvested from mature fruits, collected from populations in Wanlu Park, Hainan, China (110.313766°E, 20.033727°N). The seeds were sown in trays filled with a 2:1 peat and vermiculite (v/v) mixture, 2 months after germination. Once the second true leaves were fully developed, the seedlings were transplanted into full-strength Hoagland’s solution (Hoagland and Arnon, 1950) containing NaCl at final concentrations of 0, 1.0, 2.0, and 3.0%.

The seedlings were pre-cultured in a full-strength Hoagland’s solution with varying salt percentages for 7 days before treatment and then planted in containers. The four treatments in this experiment were as follows: (1) No NaCl (control); (2) 1.0% NaCl; (3) 2.0% NaCl; and (4) 3.0% NaCl. NaCl was added to the water nutrient solution. Each treatment consisted of three containers arranged in a randomized block (six seedlings per container). The nutrient solution was replaced every 3 days. Growth and biochemical indicators were examined at 5, 25, and 40 d after treatment. After 40 days, the transcriptomic analysis samples were analyzed. Liquid nitrogen was used to freeze the plant material, which was then stored at –80°C for downstream analysis. Three biological replicates were prepared for each sample.

### Determinations of growth, electrolyte leakage, and root activity

Growth indexes were measured using a ruler, and the increment in growth indexes was calculated using the value of every sampling day from 0 day treatment. Using the method described by Biswas et al. (2012), electrolyte leakage was determined. A method described by Yang et al. (2016b) was used to determine root activity.

### Determinations of proline, malonaldehyde, antioxidant enzyme activities, and chlorophyll content

Proline and MDA contents were determined according to a previous study (Ali et al., 2019). To assay SOD (EC 1.15.1.1) activity, the inhibition of photochemical reduction of nitro-blue tetrazolium was monitored as described by a previous study (El-Shabrawi et al., 2010). According to a previous description, POD (EC1.11.1.7.) (Zhou et al., 2017) and CAT (EC 1.11.1.6.) activity (Hasanuzzaman et al., 2011) were measured. Chlorophyll content was measured using methods described in a previous study (Lichtenthaler, 1987).

### RNA extraction

Total RNA was extracted from leaf and flower tissues using TRIzol^®^ Life Reagent (Invitrogen, Carlsbad, CA, United States) according to manufacturer’s instructions. Following the extraction, remaining genomic DNA was removed using DNase I (TaKara, United States). RNA quality was assessed using a 2100 Bioanalyzer (Agilent Technologies Inc., Santa Clara, CA, United States). RNA was quantified using a ND-2000 spectrophotometer (NanoDrop Thermo Scientific, Wilmington, DE, United States). Only high-quality RNA samples (OD_260_/_280_ = 1.8–2.2, OD_260_/_230_ ≥ 2.0, RIN ≥ 8.0, and 28S:18S ≥ 1.0, > 1 μg) were used to construct the sequencing library.

### Library preparation and Illumina NovaSeq 6000 sequencing

RNA purification, reverse transcription, and library construction and sequencing experiments were performed at Shanghai Majorbio Bio-Pharm Biotechnology Co., Ltd. (Shanghai, China). Each of these analyses followed manufacturer’s instructions (Illumina, San Diego, CA, United States). The RNA-seq libraries for each sample (Control_1, Control_2, Control_3, 3.0% NaCl_1, 3.0% NaCl_2, and 3.0% NaCl_3) were prepared using the Illumina TruSeq RNA sample preparation kit. Purified poly (A) mRNA was obtained using oligo-dT-attached magnetic beads and a fragmentation buffer. These short fragments were used as templates for the synthesis of double-stranded cDNA with the SuperScript Double-stranded cDNA Synthesis kit (Invitrogen) and random hexamer primers (Illumina). End repair was performed on the synthesized cDNA, followed by phosphorylation and the addition of “A” bases as per Illumina’s library construction protocol. cDNA target fragments of 200–300 bp were selected for using 2% Low Range Ultra Agarose and PCR amplification (15 cycles) with Phusion DNA polymerase (New England Biolabs, Boston, MA, United States). After quantification by TBS380, six RNA-seq libraries were sequenced using an Illumina NovaSeq 6000 sequencer using one lane and 2 × 150 bp paired-end reads. The raw reads generated for this study were deposited in the NCBI database under the accession number PRJNA842495.

### *De novo* assembly and annotation

*De novo* assembly was performed on the cleaned data using Trinity (Grabherr et al., 2011). BLASTX with an *e*-value of lower than 1.0 × 10^–5^ was used to predict the unigenes from the protein non-redundant (NR), Clusters of Orthologous Groups (COG), Kyoto Encyclopedia of Genes and Genomes (KEGG), and NCBI databases. The BLAST2GO program was used to obtain gene ontology (GO) annotations of uniquely assembled transcripts to describe potentially associated biological processes, molecular functions, and cellular components (Conesa et al., 2005). The KEGG was used for the metabolic pathway analysis (Ogata et al., 1999).

### Differentially expressed genes identification and functional enrichment

DEGs of two different samples were identified by calculating the expression levels of each transcript using the transcripts per million reads method. Gene abundance was calculated using RSEM (Li and Dewey, 2011). EdgeR (Robinson et al., 2010) was used to analyze gene expression and DEGs with a | log2FC| > 1 and *Q*-value ≤ 0.05. GO and KEGG analyses were enriched in DEGs to identify relevant pathways and functions (Xie et al., 2011). A Bonferroni-corrected *P*-value ≤ 0.05 was used to compare the background comprising the whole transcriptome.

### Quantitative real-time PCR analysis

Quantitative real-time PCR analysis was performed as previously described by Zhang et al. (2020b). Primer Premier 5.0 was used to create the primers (Supplementary Table 1). The length of the amplified PCR products ranged from 80 to 400 bp. The relative expression levels of the genes in various samples were estimated using the 2^–ΔΔCt^ method (Livak and Schmittgen, 2001), using actin as a reference to calculate the relative expression of the DEGs.

### Statistical analysis

Data from each group were analyzed individually using SPSS software (version 19.0). Tukey’s test value of *P* < 0.05 indicated statistical significance, and significant differences are indicated by different letters above bars.

## Results

### Effects of salt stress on morphological and physiological responses

**FIGURE 1:**
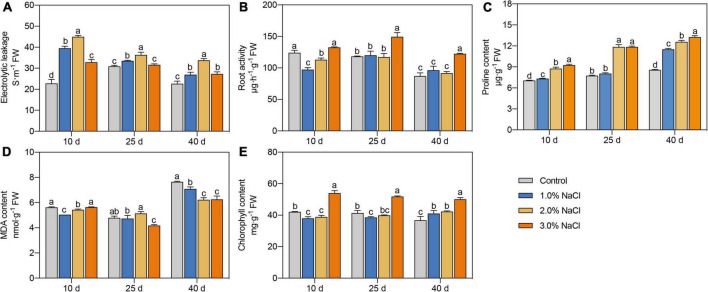
Effects of different concentrations of NaCl on electrolyte leakage **(A)**, root activity **(B)**, proline content **(C)**, MDA content **(D)**, and chlorophyll content **(E)** of *S. caseolaris* seedlings. Values are means ± SD (*n* = 3). Values with a different letter within a sampling date are significantly different (*P* < 0.05).

The increments in plant height, stem diameter, leaf length, and leaf width were higher in the 1.0, 2.0, and 3.0% NaCl treatment groups than in the 0% NaCl treatment group on days 10, 25, and 40 (Table 1). The increase in plant height, stem diameter, leaf length, and leaf width reached a maximum under the 2.0% NaCl treatment on days 10 and 25. The electrolyte leakage was significantly increased by 9.41–73.49%, 17.60–97.58%, and 20.61–44.15% under 1.0, 2.0, and 3.0% NaCl treatment groups, respectively, compared with that in the control, except on day 25 under 3.0% NaCl treatment (Figure 1A). The electrolyte leakage reached a maximum under the 2.0% NaCl treatment on days 10, 25, and 40. Root activity was significantly increased and reached a maximum (1.17–1.36-fold) under 3.0% NaCl treatment, compared with that under the 1.0 and 2.0% NaCl treatments (Figure 1B). Compared with the control, the 1.0 and 2.0% NaCl treatments significantly decreased root activity on day 10. These results indicated that 3.0% NaCl treatment increased electrolyte leakage and root activity to maintain plant growth in *S. caseolaris* seedling leaves.

**TABLE 1:** Effects of different concentration salt stress on the increment in growth indexes of *S. caseolaris* seedlings.

Treatment	Time	Plant height (cm)	Stem diameter (cm)	Leaf length (cm)	Leaf width (cm)
Control	10 days	2.37 ± 0.06d	0.89 ± 0.01c	0.82 ± 0.03c	0.32 ± 0.03c
1.0% NaCl		3.27 ± 0.12b	1.48 ± 0.09ab	1.22 ± 0.03b	0.53 ± 0.04b
2.0% NaCl		4.00 ± 0.10a	1.56 ± 0.08a	1.32 ± 0.03a	0.62 ± 0.03a
3.0% NaCl		2.70 ± 0.20c	1.37 ± 0.01b	0.62 ± 0.03d	0.28 ± 0.03c
Control	25 days	6.28 ± 0.11ab	1.12 ± 0.01a	1.05 ± 0.01c	0.22 ± 0.03b
1.0% NaCl		7.05 ± 0.63a	1.60 ± 0.14a	1.00 ± 0.01d	0.78 ± 0.04a
2.0% NaCl		7.60 ± 0.57a	1.63 ± 0.02a	1.15 ± 0.01a	0.83 ± 0.04a
3.0% NaCl		5.40 ± 0.57b	1.14 ± 0.37a	1.10 ± 0.01b	0.18 ± 0.03b
Control	40 days	7.37 ± 0.15c	1.56 ± 0.05c	0.23 ± 0.04c	0.22 ± 0.03c
1.0% NaCl		9.25 ± 0.07b	1.75 ± 0.03b	1.03 ± 0.03b	0.30 ± 0.01b
2.0% NaCl		10.50 ± 0.01a	2.11 ± 0.04a	1.10 ± 0.01a	0.73 ± 0.03a
3.0% NaCl		7.33 ± 0.35c	1.55 ± 0.03c	1.02 ± 0.03b	0.20 ± 0.01c

Values are means ± SD (n = 3). Values with a different letter within a sampling date are significantly different (P < 0.05).

Proline content significantly increased with increasing NaCl concentration on days 10 and 40 (Figure 1C). Compared with that in the control, proline content in the 3.0% NaCl treated seedlings was significantly increased by 31.01–54.90% on days 10–40. Compared with the 1.0% NaCl treatment groups, the 2.0 and 3.0% NaCl treatments significantly increased proline content by 19.36–47.38% and 15.32–47.38%, respectively, during all sample dates. Compared with the control, 1.0 and 2.0% NaCl treatment significantly decreased MDA content by 10.34 and 7.32% and 3.45 and 18.59% in leaves on days 10 and 40, respectively, while 3.0% NaCl treatment significantly decreased MDA content by 12.81 and 18.17% on days 25 and 40, respectively (Figure 1D). Compared with that in the control, the chlorophyll content was significantly decreased by 9.75% on day 10 and 6.81% on day 25 under 1.0% NaCl treatments and by 7.61% on day 10 under 2.0% NaCl treatments (Figure 1E). The chlorophyll content in the 3.0% NaCl treatment groups was significantly increased by 25.34–36.36% than in the control during all sample dates and was higher in the 3.0% NaCl-treated seedlings than that in other treated seedlings. These results indicate that 3.0% NaCl treatment can maintain higher proline and chlorophyll contents and lower MDA content in *S. caseolaris* seedling leaves.

### Antioxidant enzyme activities in response to salt stress

**FIGURE 2:**
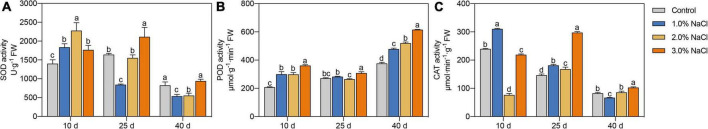
**(A–C)** Effects of 0, 1.0, 2.0, and 3.0% NaCl on antioxidant enzyme activities of *S. caseolaris* seedlings. Values are means ± SD (*n* = 3). Values with a different letter within a sampling date are significantly different (*P* < 0.05).

Different NaCl treatments were used to determine the activities of SOD, POD, and CAT in the leaves (Figures 2A–C). Compared with the control, the 1.0% NaCl-treated seedlings showed a 31.15% increase in SOD activity at day 10 and a 48.54 and 34.73% decrease at days 25 and 40, respectively. Compared with the control, 2.0% NaCl -treated seedlings showed a 62.59% increase in SOD activity at day 10 and a 32.84% decrease at day 40, whereas 3.0% NaCl treatment significantly increased SOD activity by 26.00 and 28.77% at days 10 and 25, respectively.

Compared with that in the control, POD activity was significantly increased by 44.23 and 43.47% at 10 days and 27.13 and 38.39% at 40 d under 1.0 and 2.0% NaCl treatments, respectively (Figure 2B). POD activity in 3.0% NaCl-treated seedlings compared with that in the control was increased by 13.63–73.52% on all sample days.

CAT activity was significantly increased by 29.58% at 10 days and 24.04% at 25 days and decreased by 23.34% at 40 days under 1.0% NaCl treatment compared with that in the control (Figure 2C). CAT activity in the 2.0% NaCl treated seedlings, compared with that in the control, was significantly increased by 14.81% at 25 days and decreased by 212.14% at 10 days. CAT activity was increased by 103.27% at 25 days and 24.08% at 40 days and decreased by 9.31% at 10 days under 3.0% NaCl treatment compared with that under no treatment.

The activity of antioxidant enzymes was increased in different sample periods to scavenge ROS; moreover, higher SOD, POD, and CAT activities in *S. caseolaris* seedling leaves were found at the end (40 days) of the sample dates under 3.0% NaCl treatment compared with that under other treatments.

### RNA sequencing, *De novo* assembly, and transcriptome annotation

Compared with the control, *S. caseolaris* seedling leaves at 40 d of the sample dates under 3.0% NaCl treatment had higher root activity, proline and chlorophyll content, SOD, POD, and CAT activities, and lower electrolyte leakage and MDA content. There was higher salt adaptability in *S. caseolaris* seedling under 3.0% NaCl treatment for 40 days of the treatment period compared with the 1.0 and 2.0% NaCl treatments. Thus, we performed a transcriptomic analysis of the leaves of *S. caseolaris* seedlings following the control and 3.0% NaCl treatment at 40 days. In each 3.0% NaCl and control treatment group, three biological replicates were sequenced, and 43.21 Gb of data from six cDNA libraries were analyzed. After removing the low-quality reads and adapters, 294,006,546 clean reads with a Q30 higher than 93.89% were obtained (Supplementary Table 2). The RNA-seq reads were aligned to *S. caseolaris* using Trinity software (Grabherr et al., 2011), because the reference genome sequence was not available. There were 104,392 transcripts and 47,852 unigenes, with an average length of 1,314.53 bp and an N50 length of 2,508 bp (Table 2). Unigenes with lengths of 200–500, 501–1,000, 1,001–2,000, and > 2,000 bp accounted for 41, 18, 17, and 24%, respectively (Supplementary Table 3), while 19,594 unigenes had lengths > 1,000 bp.

**TABLE 2:** Evaluation of the assembly result.

Type	Unigene	Transcript
Total number	47,852	104,392
Total base	62,902,906	183,884,555
Largest length (bp)	16,914	16,914
Smallest length (bp)	201	201
Average length (bp)	1314.53	1761.48
N50 length (bp)	2,508	2,584
E90N50 length (bp)	3,163	2,670
Fragment mapped percent (%)	66.556	87.082
GC percent (%)	44.51	44.77
TransRate score	0.30057	0.3922
BUSCO score	C:65.4% [S:61.9%; D:3.5%]	C:92.2% [S:41.1%; D:51.1%]

The 26,498 unigenes (56.04%) matched at least once with known genes at in the listed databases. Unigenes of 25,984 (54.95%) and 23,063 (48.77%) were the best hits in the NR and COG databases, respectively, followed by 21,621 (45.72%) in the GO database, 20,677 (43.73%) in the Swiss-port database, 20,220 (42.76%) in the Pfam database, and 12,023 (25.43%) in the KEGG database (Table 3).

**TABLE 3:** Statistics of annotation results.

Type	Exp_unigene number (percent)	Exp_transcript number (percent)	All_unigene number (percent)	All_transcript number (percent)
NR	25,984 (0.5495)	78,003 (0.7605)		
COG	23,063 (0.4877)	70,902 (0.6913)	21,711 (0.4537)	67,543 (0.647)
GO	21,621 (0.4572)	66,379 (0.6472)	12,095 (0.2528)	39,978 (0.383)
Swiss-Prot	20,677 (0.4373)	64,630 (0.6301)	23,159 (0.484)	72,146 (0.6911)
Pfam	20,220 (0.4276)	62,538 (0.6097)	26,091 (0.5452)	79,338 (0.76)
KEGG	12,023 (0.2543)	39,207 (0.3823)	20,765 (0.4339)	65,795 (0.6303)
Total_anno	26,498 (0.5604)	78,623 (0.7666)	26,635 (0.5566)	79,989 (0.7662)
Total	47,287 (1)	102,564 (1)	47,852 (1)	104,392 (1)

**FIGURE 3:**
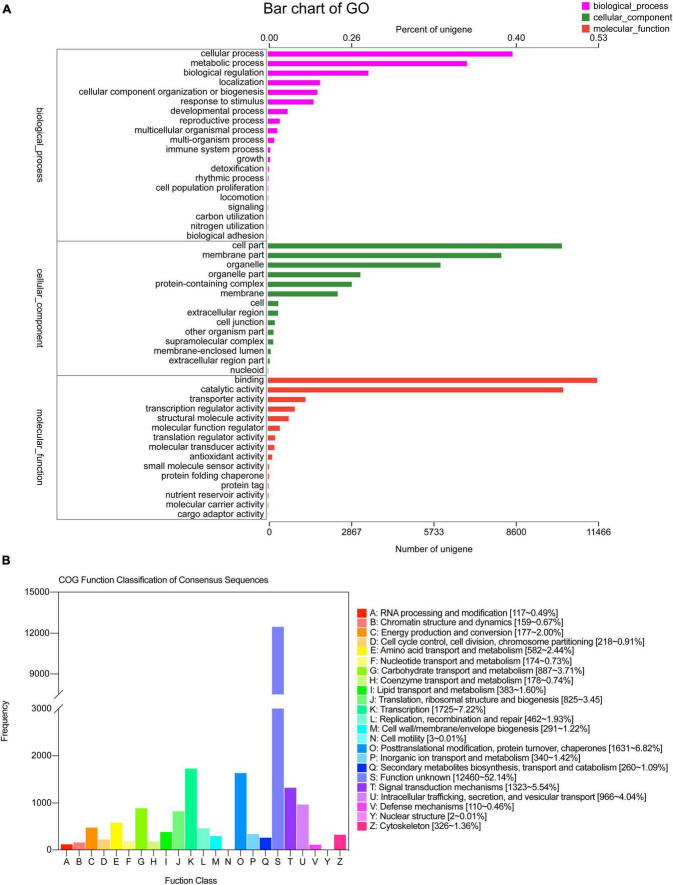
GO and COG classification statistics. **(A)** GO, **(B)** COG.

The greatest hits in the GO database were 21,621 unigenes, which were enriched for 52 GO terms categorized as biological processes, cellular components, and molecular functions. In biological processes, most of the unigenes were enriched for “cellular process” (8,520), “metabolic process” (6,931), “biological regulation” (3,502), “localization” (1,816), “cellular component organization or biogenesis” (1,726), and “response in stimulus” (1,592) terms, which accounted for 39.41, 32.06, 16.20, 8.40, 7.98, and 7.36%, respectively. The other two processes were responsible for less than 10% of the gene enrichment. For the cellular components, the largest subcategories were “cell part” (10,242; 47.37%), “membrane part” (8,130; 37.60%), “organelle” (6,013; 27.81%), “protein-containing complex” (2,921; 13.51%), and “membrane” (2,433; 11.25%). Regarding molecular function, most of the unigenes were enriched for “binding” (11,466; 53.03%) and “catalytic activity” (10,284; 47.56%) (Figure 3A).

**FIGURE 4:**
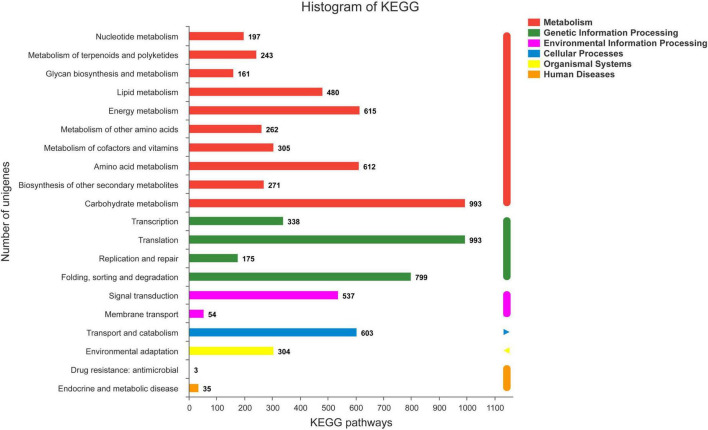
KEGG classification statistics.

The COG database was used to annotate 23,063 unigenes, which were grouped into 21 categories (Figure 3B). The following three classes with > 1,000 unigenes were identified: (K) “Transcription” (1,725, 7.22%), (O) “Posttranslational modification, protein turnover, chaperones” (1,631, 6.82%), and (T) “Signal transduction mechanisms” (1,323, 5.54%), except (S) “Function unknown.” A total of 12,023 unigenes of *S. caseolaris* were assigned to 20 KEGG categories (Figure 4). Most of the unigenes were categorized as carbohydrate metabolism (993), translation (993), folding, sorting, and degradation (799), energy metabolism (615), and amino acid metabolism (612).

### Analysis of differentially expressed genes and gene co-expression clusters

**FIGURE 5:**
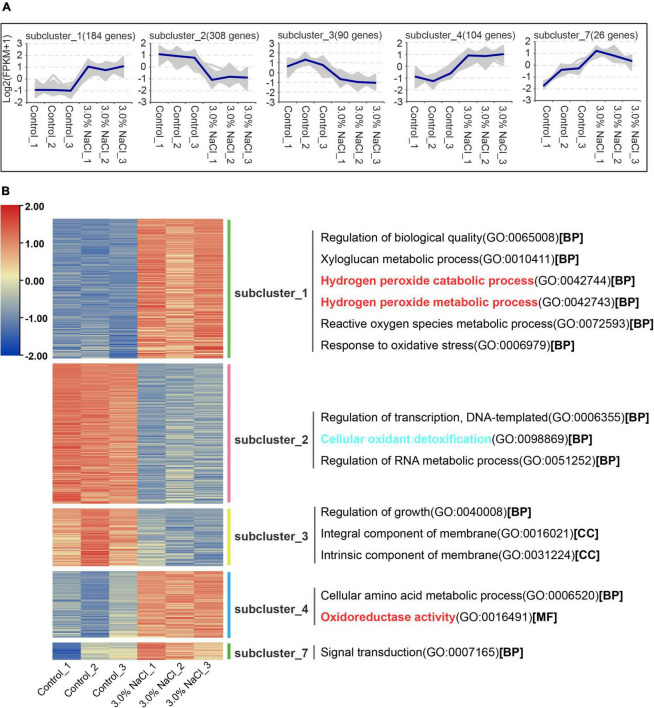
Gene co-expression clusters and heatmap analysis of DEGs in *S. caseolaris* under 3.0% NaCl treatment. **(A)** Gene number of co-expression clusters and these gene expression patterns, **(B)** heatmap and GO terms related to clusters of enriched gene co-expression.

RNA-seq reads from 3.0% NaCl and control were aligned to an average mapping rate of 87.76% in *S. caseolaris* (Supplementary Table 2). The Pearson’s correlation coefficient between the three biological replicates of the 3.0% NaCl treatment or control was approximately 0.918–1.0, which had a strong correlation (Supplementary Figure 1). The control and 3.0% NaCl treatment groups had distinct transcriptome characteristics, according to the principal component analysis (Supplementary Figure 2). This result indicated that 3.0% NaCl stress significantly affected transcriptome-wide gene expression in *S. caseolaris*. A total of 1263 DEGs in the control vs. the 3.0% NaCl treatment group were separated into ten clusters of gene co-expression patterns (Supplementary Datasets 1, 2). Clusters 1, 4, and 7 showed significantly higher gene expression in the 3.0% NaCl group compared with that in the control group, with 184, 104, and 26 genes being upregulated, respectively (Figure 5A and Supplementary Dataset 1). Based on the GO analysis, the upregulated genes were associated with the regulation of biological quality, xyloglucan metabolic process, hydrogen peroxide catabolic process, hydrogen peroxide metabolic process, ROS metabolic processes, response to oxidative stress, cellular amino acid metabolic processes, oxidoreductase activity, and signal transduction. Compared with those in the control, the genes in Cluster 2 (308) and Cluster 3 (90) were significantly downregulated in the 3.0% NaCl treatment (Supplementary Dataset 2). These genes were associated with transcriptional regulation, DNA-templated formation, cellular oxidant detoxification, regulation of RNA metabolic processes, growth regulation, integral membrane components, and intrinsic membrane components (Figure 5B).

**FIGURE 6:**
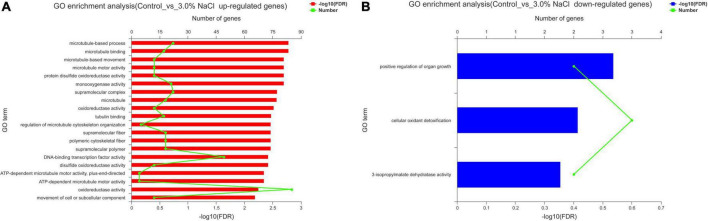
GO enrichment analysis of the DEGs in *S. caseolaris* under 3.0% NaCl treatment. **(A)** Upregulated genes, **(B)** downregulated genes.

The control and 3.0% NaCl unigenes were compared using GO enrichment analysis. The genes (859) in the control vs. the 3.0% NaCl were significantly upregulated and were enriched in several processes related to microtubules, such as microtubule-based process (GO:0007017), microtubule binding (GO:0008017), microtubule-based movement (GO:0007018), and microtubule motor activity (GO:0003777). Notably, many pathways related to oxidoreductase activity were significantly enriched, such as protein disulfide oxidoreductase activity (GO:0015035), monooxygenase activity (GO:0004497), oxidoreductase activity, with NAD(P)H as a donor, incorporation of one atom of oxygen (GO:0016709), disulfide oxidoreductase activity (GO:0015036), and oxidoreductase activity (GO:0016491) (Figure 6A). Downregulated genes (404) were significantly enriched in the positive regulation of organ growth (GO:0046622), cellular oxidant detoxification (GO:0098869), and 3-isopropylmalate dehydratase activity (GO:0003861) (Figure 6B).

### Responses of transcription factors, plant phytohormones, and antioxidant activity to salt stress

Eight candidate DEGs (four upregulated and four downregulated genes) were randomly selected, and their expression levels were measured using qRT-PCR with specific primers to validate the reliability of the RNA-seq data of *S. caseolaris* after salt stress (Supplementary Table 1). The DEGs identified in *S. caseolaris* after salt stress had expression patterns that were highly comparable with the RNA-seq data (Supplementary Figure 3), indicating that the RNA-seq data were extremely reliable. The result showed that the identified DEGs were appropriate for further research on *S. caseolaris* under salt stress.

**FIGURE 7:**
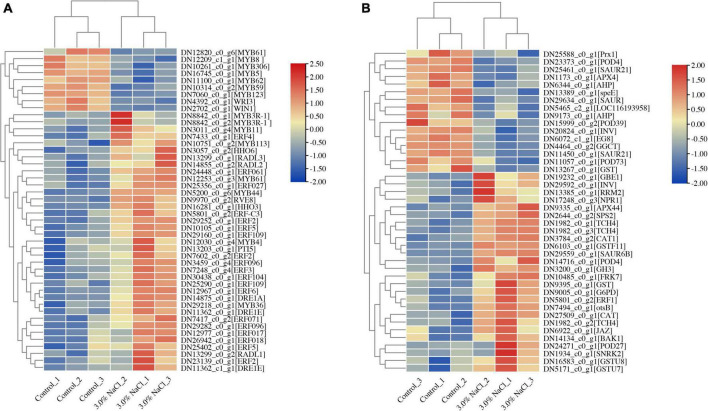
Heatmaps of salt stress-responsive genes in *S. caseolaris*. **(A)** AP2/ERF and MYB Transcription factors, **(B)** plant phytohormones and antioxidant activity related genes.

**FIGURE 8:**
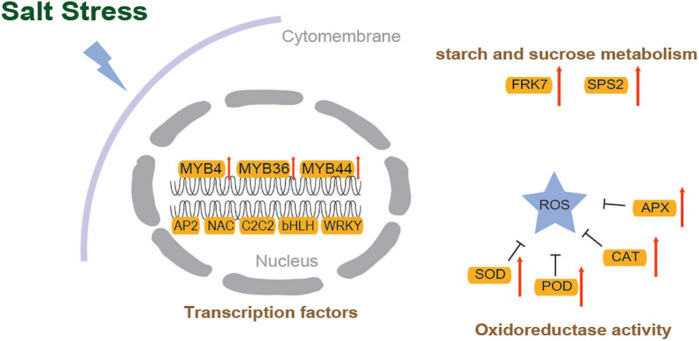
The molecular mechanism for salinity tolerance of *S. caseolaris*.

Plant growth and development depend on transcription factors (TFs) as well as plant responses to abiotic stress (Ritonga et al., 2021). In the assembled transcriptome of *S. caseolaris*, 1280 TF members were predicted. More than 60 members were identified in the MYB, AP2/ERF, C2C2, NAC, bHLH, WRKY, and GRAS families (Supplementary Figure 4). In the DEGs of the control vs. the 3.0% NaCl, 108 highly identified TFs were identified (70 upregulated and 38 downregulated), including 25 AP2 TF genes (23 upregulated and two downregulated) (Figure 7A), 21 MYB TFs (14 upregulated and 7 downregulated) (Figure 7A), 16 NAC TFs (3 upregulated and 13 downregulated), 10 C2C2 TFs (5 upregulated and 5 downregulated), 7 bHLH TFs (4 upregulated and 3 downregulated), 7 WRKY TFs (upregulated), 4 MADS-box TFs (3 upregulated and 1 downregulated), 4 GRAS TFs (3 upregulated and 1 downregulated), and other TFs (Supplementary Dataset 3). In the leaves of *S. caseolaris* after salt stress, DEGs that were associated with phytohormone transport or synthesis, such as ethylene-response factor ERF1 and auxin-responsive protein SAURs, were also identified (Figure 6B and Supplementary Dataset 4). One DEG related to the ethylene response factor was upregulated, and auxin-responsive protein SAURs were downregulated. The DEGs related to antioxidant activity, such as CAT, POD, and APX, were identified in *S. caseolaris* under salt stress (Figure 7B). Three peroxidase (POD) genes and one peroxiredoxin (Prx) gene were downregulated, and two catalase (CAT) genes, two POD (*POD4* and *POD 27*) and one L-ascorbate peroxidase (APX) gene were upregulated (Figures 7B, 8).

Following salt stress, DEGs identified in starch and sucrose metabolism (ko00500), such as probable fructokinase (FRK7) and sucrose-phosphate synthase (SPS2), were significantly upregulated in *S. caseolaris* (Figures 7B, 8). Among the DEGs, five glutathione S-transferase genes (GSTs) were found to be involved in glutathione metabolism (ko00480). These results indicate that redox balance is important for the salt stress response of *S. caseolaris*.

## Discussion

Plants respond to abiotic stresses through a series of biological processes such as transcriptional regulation, physiological responses, and biochemical metabolic processes. Salinity is a major abiotic stressor that causes osmotic stress, ion toxicity, and oxidative stress, all of which endanger plant growth and development and can lead to plant death (Zhou et al., 2017; Parmar et al., 2020). In model plants and staple crops, much research has focused on the mechanisms of salt signal perception and transcriptional regulatory pathway (Golldack et al., 2014; Yang et al., 2016b; Yang and Guo, 2018). To better identify candidate genes and understand the molecular basis of salt tolerance for improving crops, it is essential to investigate less common plants, such as mangroves and other plants with high salt tolerance. As a mangrove plant, *S. caseolaris* formed a set of salt-tolerance mechanisms that adapt to different salinities because of long-term growth in the intertidal zone of the marine environment (Tatongjai et al., 2021). It is important to identify novel salt tolerance genes and investigate the molecular mechanisms of its acclimation to salt stress (Zafar et al., 2020).

### Physiological and transcriptome level analysis of *Sonneratia caseolaris* response to salt stress

Osmotic stress and ion toxicity caused by salt stress can cause a series of secondary stresses such as oxidative stress, which significantly increase ROS in plants. Mangrove plants, including *S. caseolaris*, have a certain adaptability to salt stress, with high antioxidant capacity and a rapid response mechanism to oxidative stress (Wang et al., 2014). Antioxidant capacity confers stable adaptation to adverse environments and improves stress resistance in mangrove plants (Sur et al., 2016). Plants eliminate excess ROS formation by salt-induced stress by initiating the enzymatic system (Gill and Tuteja, 2010).

When plants were exposed to salt stress, many genes were significantly enriched in DEGs in the plant transcriptome (Xu et al., 2021), which were also found to be associated with oxidation-reduction and hydrogen peroxide catabolic processes in *S. caseolaris*. Based on the GO enrichment analysis, these genes were involved in various molecular functions, including protein disulfide oxidoreductase activity, monooxygenase activity, and oxidoreductase activity. CAT, POD, APX, and Prx enzymes are required to maintain redox homeostasis when plants are exposed to salt stress. The cumulative oxidized Prxs may suggest that cellular redox homeostasis is disrupted (Poynton and Hampton, 2014). This study found that under salt stress, many genes encoding PODs and APXs were upregulated (Figure 8), and Prx was significantly downregulated. The high expression levels of PODs and APXs contributed to salt tolerance in *S. caseolaris*.

As an antioxidant, glutathione may scavenge ROS and protect plants from oxidative stress (Zhou et al., 2018). Overexpression of the GST gene increases tobacco development during exposure to salt stress (Du et al., 2019). Similarly, in this study, numerous GST genes expression levels were found to be significantly higher after salt stress treatment. Plants produce osmotic adjustment substances such as proline, amino acids, and sugars via various metabolic pathways to reduce the damage caused by oxidative and osmotic stress from increasing salinity (Gul et al., 2022). The amount of proline significantly increased due to salt stress in *S. caseolaris*, which in previous results showed that the concentration of proline progressively increased with the time of exposure to NaCl (Naliwajski and Skłodowska, 2021). Following salt stress, the DEGs of the *S. caseolaris* transcriptome were significantly enriched in genes involved in a variety of metabolic pathways, including ROS metabolic process, xyloglucan metabolic process, and starch and sucrose metabolism. In this study, salt stress was found to induce the expression of genes that regulate metabolic processes, thereby protecting plant cells from high salt stress. The sucrose phosphate synthase (SPS) gene *SPS2* and invertase gene *INV* were found to be significantly upregulated following salt stress conditions in this study. Similarly, previous studies showed that *SPS*s enhanced tolerance of tomatoes to NaCl or H_2_O_2_ stress (Duan et al., 2021), which was further confirmed in this study. In addition, sugar content and soluble acid invertase activity in sugarcane are both directly and indirectly affected by SPS overexpression (Anur et al., 2020). Collectively, at the physiological and transcriptional levels, these results strongly indicate that *S. caseolaris* can rapidly adapt to salt stress.

### Phytohormone and transcription factor responses to salt stress

Phytohormones are considered the most important endogenous compounds that regulate physiological responses and ultimately lead to salinity adaptation (Fahad et al., 2015). Auxin transport-related genes, such as *SAURs*, were downregulated under salt stress, which is in accordance with the results of a previous study in which auxin receptor gene expression was downregulated in *Arabidopsis* under salt stress (Jiang et al., 2016). The most important stress-responsive hormone, ethylene, is crucial for plant response to salt stress (Tao et al., 2015). Genes related to ethylene response and transcription factors, such as *ERFs*, were upregulated under salt stress. In *Arabidopsis*, overexpression of *ERF96*, a minor ethylene response factor, increased salt tolerance (Wang et al., 2017). In the leaves of *S. caseolaris*, the expression levels of *ERF96* were increased in response to salt stress.

Abiotic stress signaling pathways regulated by TFs are important for salt tolerance. Salt tolerance is induced by transcription factors that bind upstream of target genes and regulate the expression levels of stress-responsive genes (Fernando, 2020). Basic leucine zippers (bZIP), Apetala (AP2), basic helix-loop-helix (bHLH) myelocytomatosis oncogenes (MYC) TFs, Zn finger TF families to myeloblastosis (MYB), WRKY, ABA-binding factor (ABF) families, and growth-regulating factors (GRFs) of TFs are also involved in phytohormone signaling pathways (Golldack et al., 2014). Under salt stress, three GRF genes were upregulated in *S. caseolaris*, indicating that stem and leaf development are modulated by saline stress; however, in *Gossypium arboreum* and *G. barbadense*, the decreased expression of GRF genes was consistent with the lack of leaf withering or yellowing under salt stress (Cao et al., 2020a). In plants, MYB transcription factors are classified into four groups: 1R-, R2R3-, R1R2R3-, and 4R-MYB. R2R3-MYBs are MYB TFs that are plant-specific (Dubos et al., 2010). R2R3-MYB transcription factors play an important role in primary and secondary metabolism, development, and responses to biotic or abiotic stresses (Yanhui et al., 2006; Dubos et al., 2010). *MYB4* is involved in the regulation of stress tolerance by improving the antioxidant defense system under abiotic stress in *Arabidopsis* (Agarwal et al., 2020). Salt tolerance enhancement likely result from the overexpression of *MYB4*, *MYB36*, and *MYB44*-mediated resistance (Persak and Pitzschke, 2014; Agarwal et al., 2020; Liu et al., 2022). This study showed that *MYB4*, *MYB36*, and *MYB44* are upregulated in *S. caseolaris* under salt stress. However, *MYB5*, *MYB8*, *MYB59*, *MYB61*, *MYB62*, *MYB123*, and *MYB306* were downregulated and were primarily involved in pectin degradation, ion transport, ROS scavenging, and secondary metabolism under abiotic stress (Zhang et al., 2016; Wei et al., 2017; Cao et al., 2020b; Islam et al., 2021; Liu et al., 2021b; Lv et al., 2021; Ma et al., 2022). For example, overexpression of *CaMYB306* resulted in earlier coloration and decreased chlorophyll content in tomatoes (Ma et al., 2022). *MYB123*-overexpressing increased POD and SOD activities while decreasing H_2_O_2_, electrolyte leakage, and MDA content under salt and drought stress (Lv et al., 2021). These results were in accordance with the GO enrichment of downregulated DEGs, indicating that in *S. caseolaris* plants under salt stress, reactive oxygen scavenging mechanisms were disturbed and repressed. However, the role of these MYB-TFs in salt tolerance requires further verification (Zafar et al., 2020).

## Conclusion

Salt stress of 3.0% significantly reduced electrolyte leakage and MDA content and significantly increased chlorophyll content and root activity in *S. caseolaris*. The activities and concentrations of antioxidant enzymes and osmotic adjustment substances significantly increased to scavenge ROS and reduce the damage caused by salinity-induced oxidative stress in *S. caseolaris*. Auxin signaling genes were downregulated at the transcriptome level and played a crucial role in salt tolerance of *S. caseolaris*. In contrast, the general response to salt stress is characterized by the upregulation of genes mediated by ethylene signaling. Gene encoding antioxidant enzymes such as *PODs* and *Prx* were downregulated, and *CATs* and *APX* were upregulated in response to salinity. Antioxidants play a key role in plant salt tolerance, and MYB TFs, such as *MYB4*, *MYB36*, and *MYB44*, that function in ROS processes play a significant role. This study provides a reference for the future investigation of salt-tolerant genes and elucidates the mechanisms of salt tolerance in *S. caseolaris*.

## Data availability statement

The original contributions presented in this study are publicly available. This data can be found here: NCBI, PRJNA842495.

## Author contributions

YaZ and YiZ defined the research theme and wrote the manuscript. LW and LL designed methods and experiments, carried out the laboratory experiments, analyzed the data, and interpreted the results. SL, EZ, and YL co-designed the experiments, carried out the laboratory experiments, and discussed the analyses and interpretation. All authors contributed seen and approved the final manuscript.

## Supplementary material

Supplementary Figure 1 Heatmap and Pearson correlation coefficients for RNA-seq replicates of *S. caseolaris* under 3.0% NaCl treatment. Three biological replicates are shown.

Supplementary Figure 2 The PCA of transcriptomes of *S. caseolaris* under 3.0% NaCl treatment and control.

Supplementary Figure 3 Expression pattern validation of eight selected DEGs in *S. caseolaris* determined by RNA-seq and qRT-PCR.

Supplementary Figure 4 The assembling transcriptome of *S. caseolaris* revealed a number of different transcription factor families.

## References

[B1] Agarwal P. Baranwal V. K. Khurana P. (2019). Genome-wide analysis of bZIP transcription factors in wheat and functional characterization of a TabZIP under abiotic stress. *Sci. Rep.* 9:4608. 10.1038/s41598-019-40659-7 30872683 PMC6418127

[B2] Agarwal P. Mitra M. Banerjee S. Roy S. (2020). MYB4 transcription factor, a member of R2R3-subfamily of MYB domain protein, regulates cadmium tolerance via enhanced protection against oxidative damage and increases expression of PCS1 and MT1C in *Arabidopsis*. *Plant Sci.* 297:110501. 10.1016/j.plantsci.2020.110501 32563471

[B3] Ali A. Maggio A. Bressan R. Yun D.-J. (2019). Role and functional differences of HKT1-Type transporters in plants under salt stress. *Int. J. Mol. Sci.* 20:1059. 10.3390/ijms20051059 30823627 PMC6429402

[B4] Anur R. M. Mufithah N. Sawitri W. D. Sakakibara H. Sugiharto B. (2020). Overexpression of sucrose phosphate synthase enhanced sucrose content and biomass production in transgenic sugarcane. *Plants* 9:200. 10.3390/plants9020200 32041093 PMC7076389

[B5] Biswas P. East A. R. Hewett E. W. Heyes J. A. (2012). Increase in electrolyte leakage as a function of chilling stress and ripening of tomato. *Acta Hortic.* 945 283–290. 10.17660/ActaHortic.2012.945.37

[B6] Cao J.-F. Huang J.-Q. Liu X. Huang C.-C. Zheng Z.-S. Zhang X.-F. (2020a). Genome-wide characterization of the GRF family and their roles in response to salt stress in *Gossypium*. *BMC Genom.* 21:575. 10.1186/s12864-020-06986-0 32831017 PMC7444260

[B7] Cao Y. Li K. Li Y. Zhao X. Wang L. (2020b). MYB transcription factors as regulators of secondary metabolism in plants. *Biology* 9:61. 10.3390/biology9030061 32213912 PMC7150910

[B8] Chen S. Zhou R. Huang Y. Zhang M. Yang G. Zhong C. (2011). Transcriptome sequencing of a highly salt tolerant mangrove species *Sonneratia alba* using Illumina platform. *Mar. Genom.* 4 129–136. 10.1016/j.margen.2011.03.005 21620334

[B9] Conesa A. Gotz S. Garcia-Gomez J. M. Terol J. Talon M. Robles M. (2005). Blast2GO: a universal tool for annotation, visualization and analysis in functional genomics research. *Bioinformatics* 21 3674–3676. 10.1093/bioinformatics/bti610 16081474

[B10] Du B. An Y. Li Y. Zhang X. Song L. Guo C. (2019). Overexpression of an alfalfa glutathione S-transferase gene improved the saline-alkali tolerance of transgenic tobacco. *Biol. Open* 8:bio043505. 10.1242/bio.043505 31471294 PMC6777358

[B11] Duan Y. Yang L. Zhu H. Zhou J. Sun H. Gong H. (2021). Structure and expression analysis of sucrose phosphate synthase, sucrose synthase and invertase gene families in *Solanum lycopersicum*. *Int. J. Mol. Sci.* 22:4698. 10.3390/ijms22094698 33946733 PMC8124378

[B12] Dubos C. Stracke R. Grotewold E. Weisshaar B. Martin C. Lepiniec L. (2010). MYB transcription factors in *Arabidopsis*. *Trends Plant Sci.* 15 573–581. 10.1016/j.tplants.2010.06.005 20674465

[B13] Ebrahim W. Kjer J. El Amrani M. Wray V. Lin W. Ebel R. (2012). Pullularins E and F, two new peptides from the endophytic fungus *Bionectria ochroleuca* isolated from the mangrove plant *Sonneratia caseolaris*. *Mar. Drugs* 10 1081–1091. 10.3390/md10051081 22822358 PMC3397455

[B14] El-Shabrawi H. Kumar B. Kaul T. Reddy M. K. Singla-Pareek S. L. Sopory S. K. (2010). Redox homeostasis, antioxidant defense, and methylglyoxal detoxification as markers for salt tolerance in *Pokkali* rice. *Protoplasma* 245 85–96. 10.1007/s00709-010-0144-6 20419461

[B15] Fahad S. Hussain S. Matloob A. Khan F. A. Khaliq A. Saud S. (2015). Phytohormones and plant responses to salinity stress: a review. *Plant Growth Regul.* 75 391–404. 10.1007/s10725-014-0013-y

[B16] Feng X. Xu S. Li J. Yang Y. Chen Q. Lyu H. (2020). Molecular adaptation to salinity fluctuation in tropical intertidal environments of a mangrove tree *Sonneratia alba*. *BMC Plant Biol.* 20:178. 10.1186/s12870-020-02395-3 32321423 PMC7178616

[B17] Fernando V. C. D. (2020). “Major transcription factor families involved in salinity stress tolerance in plants,” in *Transcription Factors for Abiotic Stress Tolerance in Plants*, ed. Wani S. H. (Amsterdam: Elsevier), 99–109. 10.1016/B978-0-12-819334-1.00007-1

[B18] Gill S. S. Tuteja N. (2010). Reactive oxygen species and antioxidant machinery in abiotic stress tolerance in crop plants. *Plant Physiol. Biochem.* 48 909–930. 10.1016/j.plaphy.2010.08.016 20870416

[B19] Golldack D. Li C. Mohan H. Probst N. (2014). Tolerance to drought and salt stress in plants: unraveling the signaling networks. *Front. Plant Sci.* 5:151. 10.3389/fpls.2014.00151 24795738 PMC4001066

[B20] Gong Z. (2021). Plant abiotic stress: new insights into the factors that activate and modulate plant responses. *J. Integr. Plant Biol.* 63 429–430. 10.1111/jipb.13079 33657281

[B21] Grabherr M. G. Haas B. J. Yassour M. Levin J. Z. Thompson D. A. Amit I. (2011). Full-length transcriptome assembly from RNA-Seq data without a reference genome. *Nat. Biotechnol.* 29 644–652. 10.1038/nbt.1883 21572440 PMC3571712

[B22] Gul Z. Tang Z.-H. Arif M. Ye Z. (2022). An insight into abiotic stress and influx tolerance mechanisms in plants to cope in saline environments. *Biology* 11:597. 10.3390/biology11040597 35453796 PMC9028878

[B23] Guo C. Guo R. Xu X. Gao M. Li X. Song J. (2014). Evolution and expression analysis of the grape (*Vitis vinifera* L.) WRKY gene family. *J. Exp. Bot.* 65 1513–1528. 10.1093/jxb/eru007 24510937 PMC3967086

[B24] Hasanuzzaman M. Hossain M. A. Fujita M. (2011). Nitric oxide modulates antioxidant defense and the methylglyoxal detoxification system and reduces salinity-induced damage of wheat seedlings. *Plant Biotechnol. Rep.* 5 353–365. 10.1007/s11816-011-0189-9 21264525

[B25] Hasanuzzaman M. Raihan M. R. H. Masud A. A. C. Rahman K. Nowroz F. Rahman M. (2021). Regulation of reactive oxygen species and antioxidant defense in plants under salinity. *Int. J. Mol. Sci.* 22:9326. 10.3390/ijms22179326 34502233 PMC8430727

[B26] Hoagland D. R. Arnon D. I. (1950). The water-culture method for growing plants without soil. *Circular. California Agricultural Experiment Station* 347:32. Available online at: https://www.cabdirect.org/cabdirect/abstract/19500302257

[B27] Huang J. Lu X. Yan H. Chen S. Zhang W. Huang R. (2012). Transcriptome characterization and sequencing-based identification of salt-responsive genes in *Millettia pinnata*, a semi-mangrove plant. *DNA Res.* 19 195–207. 10.1093/dnares/dss004 22351699 PMC3325082

[B28] Islam M. Q. Hasan M. N. Hoque H. Jewel N. A. Bhuiyan M. F. H. Prodhan S. H. (2021). Characterization of transcription factor MYB59 and expression profiling in response to low K^+^ and NO_3_^–^ in indica rice (*Oryza sativa* L.). *J. Genet. Eng. Biotechnol.* 19:167. 10.1186/s43141-021-00248-6 34704216 PMC8548439

[B29] Ismail A. M. Horie T. (2017). Genomics, Physiology, and molecular breeding approaches for improving salt tolerance. *Annu. Rev. Plant Biol.* 68 405–434. 10.1146/annurev-arplant-042916-040936 28226230

[B30] Jahan B. Iqbal N. Fatma M. Sehar Z. Masood A. Sofo A. (2021). Ethylene supplementation combined with split application of nitrogen and sulfur protects salt-inhibited photosynthesis through optimization of proline metabolism and antioxidant system in mustard (*Brassica juncea* L.). *Plants* 10:1303. 10.3390/plants10071303 34199061 PMC8309136

[B31] Jiang K. Moe-Lange J. Hennet L. Feldman L. J. (2016). Salt Stress affects the redox status of *Arabidopsis* root meristems. *Front. Plant Sci.* 7:81. 10.3389/fpls.2016.00081 26904053 PMC4744855

[B32] Li B. Dewey C. N. (2011). RSEM: accurate transcript quantification from RNA-Seq data with or without a reference genome. *BMC Bioinform.* 12:323. 10.1186/1471-2105-12-323 21816040 PMC3163565

[B33] Li F.-L. Zan Q.-J. Hu Z.-Y. Shin P.-K. S. Cheung S.-G. Wong Y.-S. (2016). Are photosynthetic characteristics and energetic cost important invasive traits for *Alien Sonneratia* species in South China? *PLoS One* 11:e0157169. 10.1371/journal.pone.0157169 27286250 PMC4902315

[B34] Li Y. Zhang L. Zhu P. Cao Q. Sun J. Li Z. (2019). Genome-wide identification, characterisation and functional evaluation of WRKY genes in the sweet potato wild ancestor *Ipomoea trifida* (H.B.K.) G. Don. under abiotic stresses. *BMC Genet*. 20:90. 10.1186/s12863-019-0789-x 31795942 PMC6889533

[B35] Li M. Chen R. Jiang Q. Sun X. Zhang H. Hu Z. (2021a). GmNAC06, a NAC domain transcription factor enhances salt stress tolerance in soybean. *Plant Mol. Biol.* 105 333–345. 10.1007/s11103-020-01091-y 33155154 PMC7858558

[B36] Li Q. Gao C. Xu K. Jiang Y. Niu J. Yin G. (2021b). Transcriptome-based analysis of resistance mechanism to black point caused by *Bipolaris sorokiniana* in wheat. *Sci. Rep.* 11:6911. 10.1038/s41598-021-86303-1 33767270 PMC7994838

[B37] Li Y. Lyu Y. Huang J. Huang K. Yu J. (2021c). Transcriptome sequencing reveals high-salt diet-induced abnormal liver metabolic pathways in mice. *BMC Gastroenterol.* 21:335. 10.1186/s12876-021-01912-4 34454434 PMC8397858

[B38] Lichtenthaler H. K. (1987). [34] Chlorophylls and carotenoids: pigments of photosynthetic biomembranes. *Methods Enzymol.* 148 350–382. 10.1016/0076-6879(87)48036-1

[B39] Liu D. Li Y.-Y. Zhou Z.-C. Xiang X. Liu X. Wang J. (2021a). Tobacco transcription factor bHLH123 improves salt tolerance by activating NADPH oxidase NtRbohE expression. *Plant Physiol.* 186 1706–1720. 10.1093/plphys/kiab176 33871656 PMC8260122

[B40] Liu Z.-Y. Li X.-P. Zhang T.-Q. Wang Y.-Y. Wang C. Gao C.-Q. (2021b). Overexpression of ThMYB8 mediates salt stress tolerance by directly activating stress-responsive gene expression. *Plant Sci.* 302:110668. 10.1016/j.plantsci.2020.110668 33288032

[B41] Liu T. Chen T. Kan J. Yao Y. Guo D. Yang Y. (2022). The GhMYB36 transcription factor confers resistance to biotic and abiotic stress by enhancing PR1 gene expression in plants. *Plant Biotechnol. J.* 20 722–735. 10.1111/pbi.13751 34812570 PMC8989497

[B42] Livak K. J. Schmittgen T. D. (2001). Analysis of relative gene expression data using real-time quantitative PCR and the 2^–ΔΔCT^ method. *Methods* 25 402–408. 10.1006/meth.2001.1262 11846609

[B43] Lv K. Wei H. Liu G. (2021). A R2R3-MYB transcription factor gene, BpMYB123, regulates BpLEA14 to improve drought tolerance in *Betula platyphylla*. *Front. Plant Sci.* 12:791390. 10.3389/fpls.2021.791390 34956289 PMC8702527

[B44] Ma X. Yu Y.-N. Jia J.-H. Li Q.-H. Gong Z.-H. (2022). The pepper MYB transcription factor CaMYB306 accelerates fruit coloration and negatively regulates cold resistance. *Sci. Hortic.* 295:110892.

[B45] Munns R. Tester M. (2008). Mechanisms of salinity tolerance. *Annu. Rev. Plant Biol.* 59 651–681. 10.1146/annurev.arplant.59.032607.092911 18444910

[B46] Naliwajski M. Skłodowska M. (2021). The relationship between the antioxidant system and proline metabolism in the leaves of cucumber plants acclimated to salt stress. *Cells* 10:609. 10.3390/cells10030609 33801884 PMC7998282

[B47] Ogata H. Goto S. Sato K. Fujibuchi W. Bono H. Kanehisa M. (1999). KEGG: kyoto encyclopedia of genes and genomes. *Nucleic Acids Res.* 27 29–34. 9847135 10.1093/nar/27.1.29 PMC148090

[B48] Parmar S. Gharat S. A. Tagirasa R. Chandra T. Behera L. Dash S. K. (2020). Identification and expression analysis of miRNAs and elucidation of their role in salt tolerance in rice varieties susceptible and tolerant to salinity. *PLoS One* 15:e0230958. 10.1371/journal.pone.0230958 32294092 PMC7159242

[B49] Persak H. Pitzschke A. (2014). Dominant repression by *Arabidopsis* transcription factor MYB44 causes oxidative damage and hypersensitivity to abiotic stress. *Int. J. Mol. Sci.* 15 2517–2537. 10.3390/ijms15022517 24531138 PMC3958865

[B50] Poynton R. A. Hampton M. B. (2014). Peroxiredoxins as biomarkers of oxidative stress. *Biochim. Biophys. Acta BBA Gen. Subj.* 1840 906–912. 10.1016/j.bbagen.2013.08.001 23939310

[B51] Robinson M. D. McCarthy D. J. Smyth G. K. (2010). EdgeR: a bioconductor package for differential expression analysis of digital gene expression data. *Bioinformatics* 26 139–140. 10.1093/bioinformatics/btp616 19910308 PMC2796818

[B52] Ritonga F. N. Ngatia J. N. Wang Y. Khoso M. A. Farooq U. Chen S. (2021). AP2/ERF, an important cold stress-related transcription factor family in plants: A review. *Physiol. Mol. Biol. Plants* 27, 1953–1968. 10.1007/s12298-021-01061-8 34616115 PMC8484489

[B53] Sachdev S. Ansari S. A. Ansari M. I. Fujita M. Hasanuzzaman M. (2021). Abiotic stress and reactive oxygen species: generation, signaling, and defense mechanisms. *Antioxidants* 10:277. 10.3390/antiox10020277 33670123 PMC7916865

[B54] Sies H. Murphy M. E. Di Mascio P. Stahl W. (1992). “Tocopherols, carotenoids and the glutathione system,” in *Lipid-Soluble Antioxidants: Biochemistry and Clinical Applications*, eds Ong A. S. H. Packer L. (Basel: Birkhäuser Basel), 160–165. 10.1007/978-3-0348-7432-8_14

[B55] Sur T. Hazra A. Hazra A. Bhattacharyya D. (2016). Antioxidant and hepatoprotective properties of Indian Sunderban mangrove *Bruguiera gymnorrhiza* L. leave. *J. Basic Clin. Pharm.* 7 75–79. 10.4103/0976-0105.183262 27330259 PMC4910471

[B56] Tanveer M. Yousaf U. (2020). “Plant single-cell biology and abiotic stress tolerance,” in *Plant Life Under Changing Environment* eds Tripathi D. K. Singh V. P. Chauhan D. K. Sharma S. Prasad S. M. Dubey N. K. (Amsterdam: Elsevier), 611–626. 10.1016/B978-0-12-818204-8.00026-6

[B57] Tao J.-J. Chen H.-W. Ma B. Zhang W.-K. Chen S.-Y. Zhang J.-S. (2015). The role of ethylene in plants under salinity stress. *Front. Plant Sci.* 6:1059. 10.3389/fpls.2015.01059 26640476 PMC4661241

[B58] Tatongjai S. Kraichak E. Kermanee P. (2021). Comparative anatomy and salt management of *Sonneratia caseolaris* (L.) Engl. (*Lythraceae*) grown in saltwater and freshwater. *PeerJ* 9:e10962. 10.7717/peerj.10962 33665038 PMC7916540

[B59] Tomlinson P. B. (1994). *The Botany of Mangroves*, 1st pbk. Edn. Cambridge: Cambridge University Press.

[B60] Wang H. Xiao X. Yang M. Gao Z. Zang J. Fu X. (2014). Effects of salt stress on antioxidant defense system in the root of *Kandelia candel*. *Bot. Stud.* 55:57. 10.1186/s40529-014-0057-3 28510976 PMC5430347

[B61] Wang X. Hou C. Zheng K. Li Q. Siyu C. Wang S. (2017). Overexpression of ERF96, a small ethylene response factor gene enhances salt tolerance in *Arabidopsis*. *Biol. Plant* 61 693–701. 10.1007/s10535-017-0734-7

[B62] Wang Y. Dai A. Tang T. (2022). Weak effect of gypsy retrotransposon bursts on *Sonneratia alba* salt stress gene expression. *Front. Plant Sci.* 12:830079. 10.3389/fpls.2021.830079 35111190 PMC8801733

[B63] Wei H. Zhao H. Su T. Bausewein A. Greiner S. Harms K. (2017). Chicory R2R3-MYB transcription factors CiMYB5 and CiMYB3 regulate fructan 1-exohydrolase expression in response to abiotic stress and hormonal cues. *J. Exp. Bot.* 68 4323–4338. 10.1093/jxb/erx210 28922763 PMC5853547

[B64] Xiao X. Hong Y. Xia W. Feng S. Zhou X. Fu X. (2016). Transcriptome analysis of *Ceriops tagal* in saline environments using RNA-sequencing. *PLoS One* 11:e0167551. 10.1371/journal.pone.0167551 27936168 PMC5147905

[B65] Xie C. Mao X. Huang J. Ding Y. Wu J. Dong S. (2011). KOBAS 2.0: a web server for annotation and identification of enriched pathways and diseases. *Nucleic Acids Res.* 39 316–322. 10.1093/nar/gkr483 21715386 PMC3125809

[B66] Xie R. Pan X. Zhang J. Ma Y. He S. Zheng Y. (2018). Effect of salt-stress on gene expression in citrus roots revealed by RNA-seq. *Funct. Integr. Genom.* 18 155–173. 10.1007/s10142-017-0582-8 29264749

[B67] Xu Y. Lu J. Zhang J. Liu D. Wang Y. Niu Q. (2021). Transcriptome revealed the molecular mechanism of *Glycyrrhiza inflata* root to maintain growth and development, absorb and distribute ions under salt stress. *BMC Plant Biol.* 21:599. 10.1186/s12870-021-03342-6 34915868 PMC8675533

[B68] Yang Y. Duke N. C. Peng F. Li J. Yang S. Zhong C. (2016a). Ancient geographical barriers drive differentiation among *Sonneratia caseolaris* populations and recent divergence from S. lanceolata. *Front. Plant Sci.* 7:1618. 10.3389/fpls.2016.01618 27833634 PMC5080369

[B69] Yang Z. Li Y. Li P. Zhang F. Thomas B. W. (2016b). Effect of difference between day and night temperature on tomato (*Lycopersicon esculentum* Mill.) root activity and low molecular weight organic acid secretion. *Soil Sci. Plant Nutr.* 62 423–431. 10.1080/00380768.2016.1224449

[B70] Yang Y. Guo Y. (2018). Unraveling salt stress signaling in plants: salt stress signaling. *J. Integr. Plant Biol.* 60 796–804. 10.1111/jipb.12689 29905393

[B71] Yang Y. Zheng C. Zhong C. Lu T. Gul J. Jin X. (2021). Transcriptome analysis of *Sonneratia caseolaris* seedlings under chilling stress. *PeerJ* 9:e11506. 10.7717/peerj.11506 34141477 PMC8180195

[B72] Yanhui C. Xiaoyuan Y. Kun H. Meihua L. Jigang L. Zhaofeng G. (2006). The MYB transcription factor superfamily of *Arabidopsis*: expression analysis and phylogenetic comparison with the rice MYB family. *Plant Mol. Biol.* 60 107–124. 10.1007/s11103-005-2910-y 16463103

[B73] Zafar S. A. Zaidi S. S.-A. Gaba Y. Singla-Pareek S. L. Dhankher O. P. Li X. (2020). Engineering abiotic stress tolerance via CRISPR/Cas-mediated genome editing. *J. Exp. Bot.* 71 470–479. 10.1093/jxb/erz476 31644801

[B74] Zelm E. Zhang Y. Testerink C. (2020). Salt tolerance mechanisms of plants. *Annu. Rev. Plant Biol.* 71 403–433. 10.1146/annurev-arplant-050718-100005 32167791

[B75] Zhang J. Shi S. Z. Jiang Y. Zhong F. Liu G. Yu C. (2021). Genome-wide investigation of the AP2/ERF superfamily and their expression under salt stress in Chinese willow (*Salix matsudana*). *PeerJ* 9:e11076. 10.7717/peerj.11076 33954030 PMC8051338

[B76] Zhang X. Chen L. Shi Q. Ren Z. (2020a). SlMYB102, an R2R3-type MYB gene, confers salt tolerance in transgenic tomato. *Plant Sci.* 291:110356. 10.1016/j.plantsci.2019.110356 31928668

[B77] Zhang Y. Chen Y. Zhou Y. Zhang J. Bai H. Zheng C. (2020b). Comparative transcriptome reveals the genes’ adaption to herkogamy of *Lumnitzera littorea* (Jack) voigt. *Front. Genet.* 11:584817. 10.3389/fgene.2020.584817 33363568 PMC7753066

[B78] Zhang X. Guo Q. Qin L. Li L. (2022). A Cys2His2 zinc finger transcription factor BpSZA1 positively modulates salt stress in *Betula platyphylla*. *Front. Plant Sci.* 13:823547. 10.3389/fpls.2022.823547 35693173 PMC9174930

[B79] Zhang Z. Hu X. Zhang Y. Miao Z. Xie C. Meng X. (2016). Opposing control by transcription factors MYB61 and MYB3 increases freezing tolerance by relieving C-repeat binding factor suppression. *Plant Physiol*. 172 1306–1323. 10.1104/pp.16.00051 27578551 PMC5047070

[B80] Zhong C. Li D. Zhang Y. (2020). Description of a new natural *Sonneratia* hybrid from Hainan island, China. *PhytoKeys* 154 1–9. 53223 10.3897/phytokeys.154 32843846 PMC7417295

[B81] Zhou Y. Diao M. Cui J. Chen X. Wen Z. Zhang J. (2018). Exogenous GSH protects tomatoes against salt stress by modulating photosystem II efficiency, absorbed light allocation and H_2_O_2_-scavenging system in chloroplasts. *J. Integr. Agric.* 17 2257–2272. 10.1016/S2095-3119(18)62068-4

[B82] Zhou Y. Wen Z. Zhang J. Chen X. Cui J. Xu W. (2017). Exogenous glutathione alleviates salt-induced oxidative stress in tomato seedlings by regulating glutathione metabolism, redox status, and the antioxidant system. *Sci. Hortic.* 220 90–101. 10.1016/j.scienta.2017.02.021

[B83] Zulfiqar F. Akram N. A. Ashraf M. (2020). Osmoprotection in plants under abiotic stresses: new insights into a classical phenomenon. *Planta* 251:3. 10.1007/s00425-019-03293-1 31776765

