# Supplementary material for: Comparative transcriptome analysis unveiling reactive oxygen species scavenging system of Sonneratia caseolaris under salinity stress
Source: Front Plant Sci. 2022 Jul 25;13:953450. doi: 10.3389/fpls.2022.953450 (PMC9358527; doi:10.3389/fpls.2022.953450)
Supplement: Supplementary file 1 [file Table_1.DOCX]

Table S1.The primer sequences of DEGs by qRT-PCR analysis.

| Gene Name | Primer sequences（5'-3') | Sequences length(bp) |
| --- | --- | --- |
| *Actin* | FP:CGCCTCTCGTCTCCTCTTCTCC RP:AGCAAATCCAGCCTTCACCATTCC | 125  125 |
| *CAT* | FP:GGCGAGACAGGTGCGTGATTAC RP:CAGAGCCTCAACCCACCGTTTG | 116  116 |
| *POD4* | FP:TTGCCTCTCCTCTTCCTCCTTCAC RP:TTGTTGTCCACTGCGTCCCTTTC | 346  346 |
| *EG8* | FP:CCTTCGTGTCCTCCTGCTCCTC RP:GTCGTAAACGCCATCGGGAACC | 258  258 |
| *ERF5* | FP:GAGTGGTTGCAGTTCAGCTCCTC RP:GGTGATGGCGACTTCTCCTTCTTG | 383  383 |
| *ERF3* | FP:GAAGAAGGCTCGTGTGTGGCTAG RP:AGGATTCAACCGTGCTGCTCATG | 230  230 |
| *GSTU7* | FP:TCCTGAACAAGAGTCCGCTCCTC RP:CTATGCTTTGTGCCCTCTCCTTCTC | 270  270 |
| *MYB8* | FP:GCTTGGACGGCAATGGAAGACC RP:TTTCCCGCACCTCCTAAGACCTG | 108  108 |
| *MYB123* | FP:GTACCTACGAGTGATGCGATGCC RP:ATGCCAGAGATTCCATGTCCAAGTC | 88  88 |
