# Supplementary material for: Comparative transcriptome analysis unveiling reactive oxygen species scavenging system of Sonneratia caseolaris under salinity stress
Source: Front Plant Sci. 2022 Jul 25;13:953450. doi: 10.3389/fpls.2022.953450 (PMC9358527; doi:10.3389/fpls.2022.953450)
Supplement: Supplementary file 2 [file Table_2.DOCX]

Table S2.[Quality control of sequencing data](https://pubmed.ncbi.nlm.nih.gov/33552473/#:~:text=Quality%20control%20is%20an%20essential%20first%20step%20in,entrenched%20in%20standard%20pipelines%20at%20most%20sequencing%20centers.).

| Sample | Clean reads | Base number | Mapped reads | Mapped ratio | Q30 | GC content |
| --- | --- | --- | --- | --- | --- | --- |
| Control_1 | 24,910,231 | 7,343,211,583 | 21,786,423 | 87.46% | 94.06% | 49.16% |
| Control_2 | 24,390,160 | 7,171,711,316 | 21,471,789 | 88.03% | 93.91% | 48.95% |
| Control_3 | 25,303,197 | 7,393,180,404 | 22,263,385 | 87.99% | 94.21% | 49.53% |
| 3.0% NaCl_1 | 25,412,053 | 7,473,725,339 | 22,364,253 | 88.01% | 94.17% | 49.84% |
| 3.0% NaCl_2 | 22,092,501 | 6,510,913,737 | 19,282,766 | 87.28% | 93.89% | 49.99% |
| 3.0% NaCl_3 | 24,895,131 | 7,312,423,009 | 21,854,966 | 87.79% | 94.03% | 49.89% |
