# Supplementary material for: Comparative transcriptome analysis unveiling reactive oxygen species scavenging system of Sonneratia caseolaris under salinity stress
Source: Front Plant Sci. 2022 Jul 25;13:953450. doi: 10.3389/fpls.2022.953450 (PMC9358527; doi:10.3389/fpls.2022.953450)
Supplement: Supplementary file 3 [file Table_3.DOCX]

Table S3.[Length distribution of unigenes.](https://www.researchgate.net/figure/Length-distribution-of-unigenes-and-transcripts_tbl2_329986513)

| Length | Number of Unigene | Percent of Unigene |
| --- | --- | --- |
| 200~500 | 19858 | 41% |
| 501~1000 | 8400 | 18% |
| 1001~1500 | 4130 | 9% |
| 1501~2000 | 3772 | 8% |
| 2001~2500 | 3318 | 7% |
| 2501~3000 | 2609 | 5% |
| 3001~3500 | 1916 | 4% |
| 3501~4000 | 1325 | 3% |
| 4001~4500 | 868 | 2% |
| >4500 | 1656 | 3% |
