# Supplementary figures and images for: Comparative transcriptome analysis unveiling reactive oxygen species scavenging system of Sonneratia caseolaris under salinity stress
Source: Front Plant Sci. 2022 Jul 25;13:953450. doi: 10.3389/fpls.2022.953450 (PMC9358527; doi:10.3389/fpls.2022.953450)

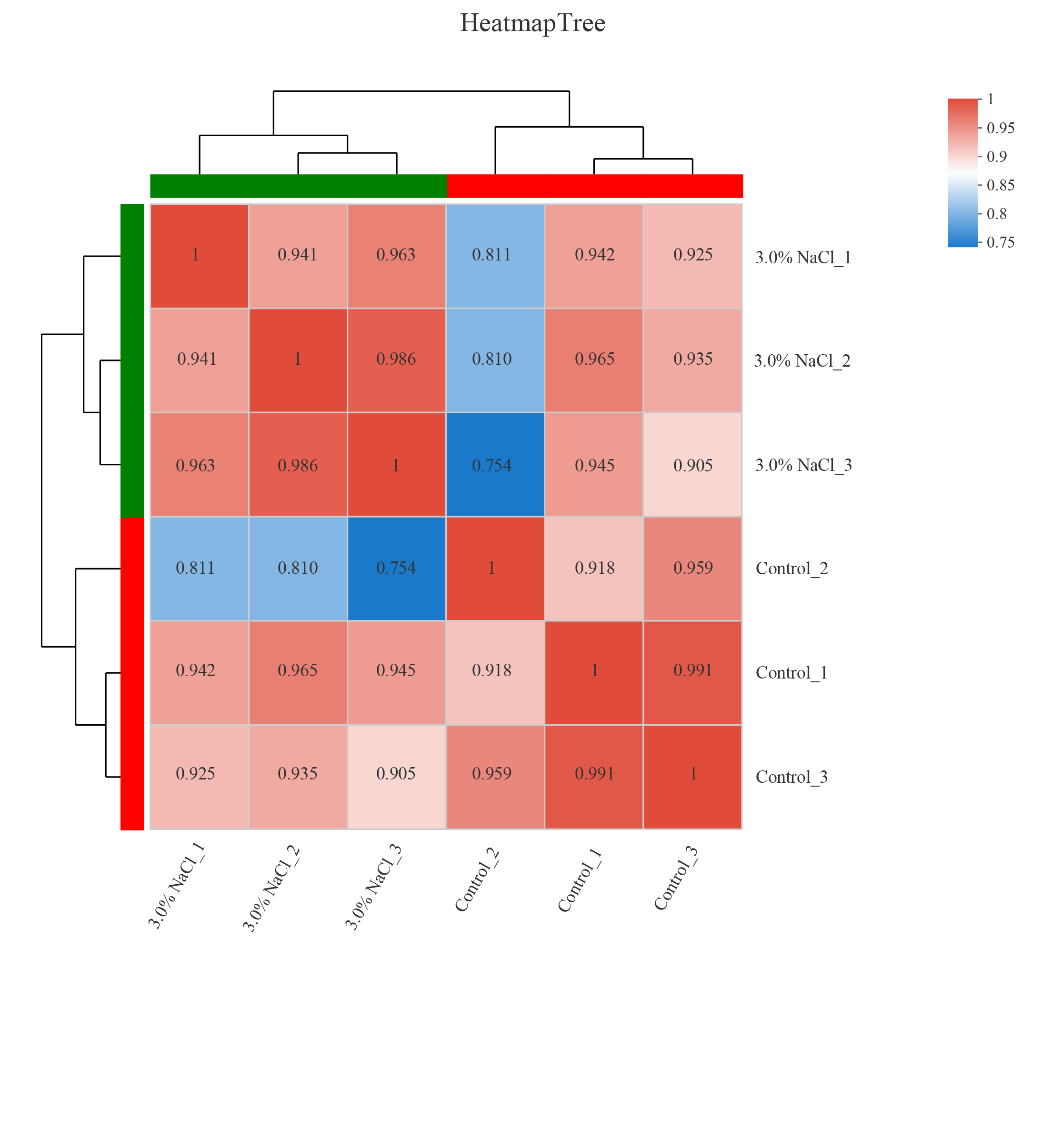

Supplement: Supplementary Figure 1 — Heatmap and Pearson correlation coefficients for RNA-seq replicates of S. caseolaris under 3.0% NaCl treatment. Three biological replicates are shown. [file Image_1.TIF]

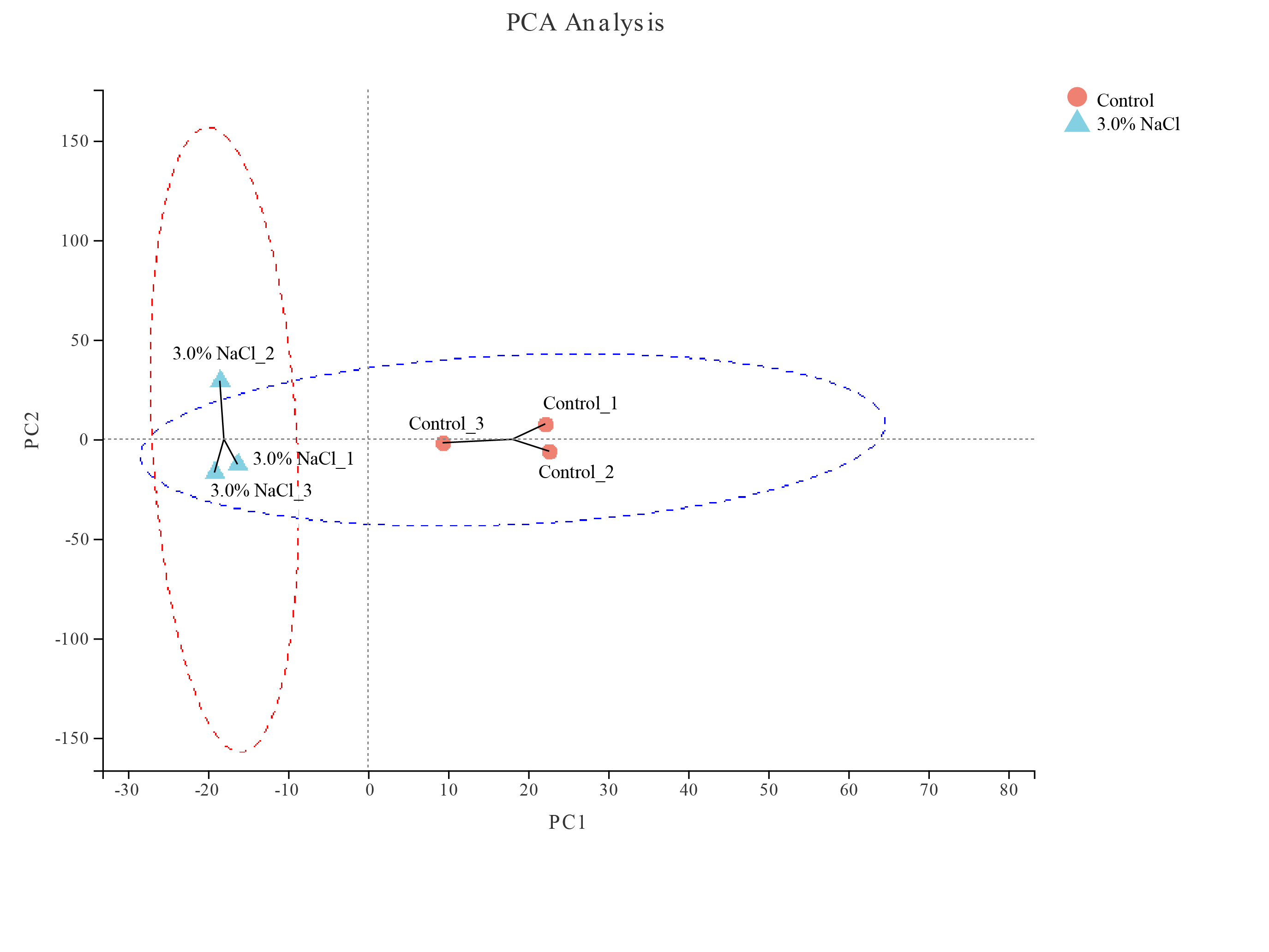

Supplement: Supplementary Figure 2 — The PCA of transcriptomes of S. caseolaris under 3.0% NaCl treatment and control. [file Image_2.TIF]

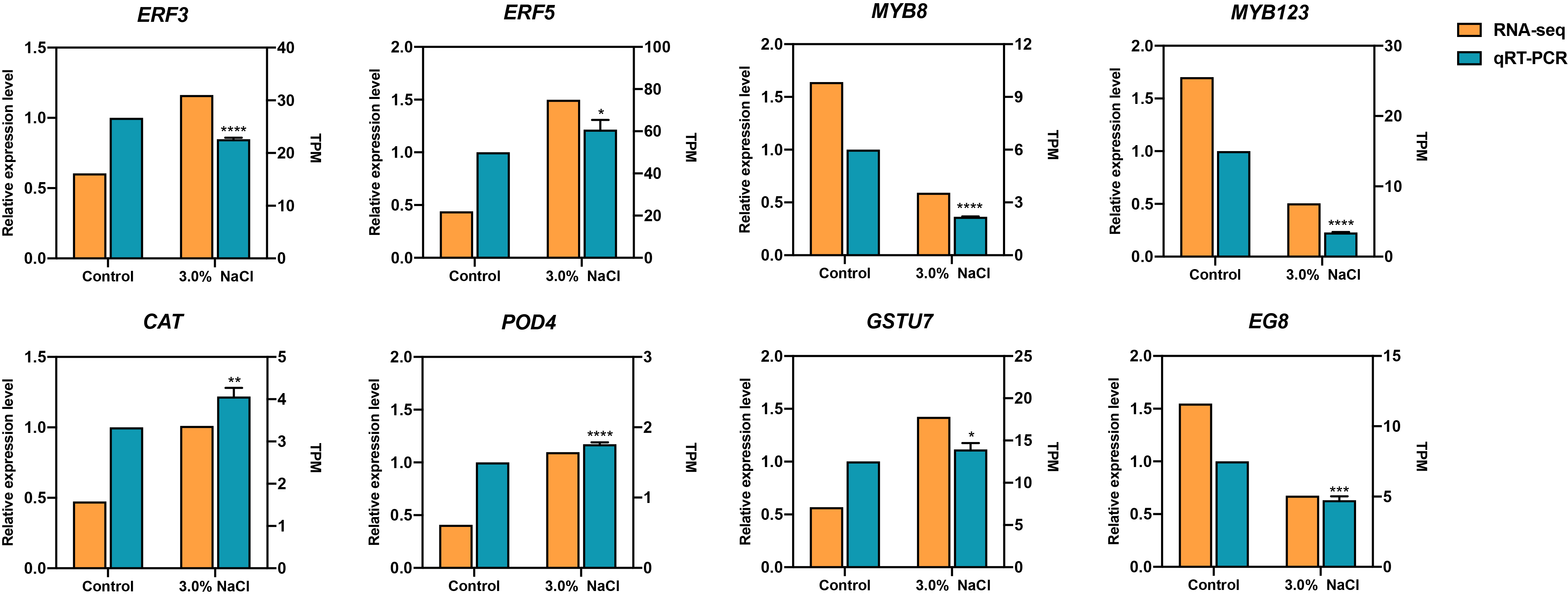

Supplement: Supplementary Figure 3 — Expression pattern validation of eight selected DEGs in S. caseolaris determined by RNA-seq and qRT-PCR. [file Image_3.TIF]

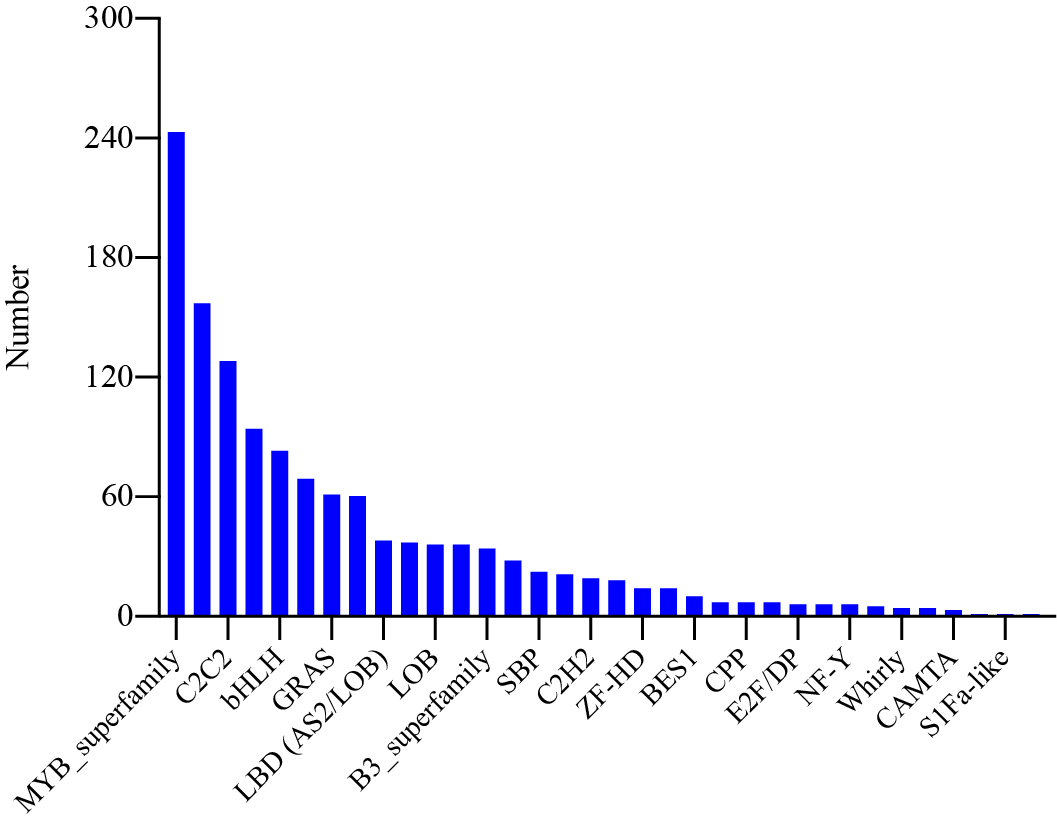

Supplement: Supplementary Figure 4 — The assembling transcriptome of S. caseolaris revealed a number of different transcription factor families. [file Image_4.TIF]
